# Impact of Reducing Fluoroscopy Pulse Rate on Adult Modified Barium Swallow Studies

**DOI:** 10.1007/s00455-023-10643-5

**Published:** 2024-01-24

**Authors:** Heather Shaw Bonilha, Erin L. Reedy, Janina Wilmskoetter, Paul J. Nietert, Bonnie Martin-Harris

**Affiliations:** 1 Health Sciences and Research, College of Health Professions, Medical University of South Carolina, 77 President Street, MSC 700, Charleston, SC 29425-2503 USA; 2 Department of Otolaryngology – Head and Neck Surgery, Medical University of South Carolina, 135 Rutledge Avenue, MSC 550, Charleston, SC 29425 USA; 3 Department of Communication Sciences and Disorders, University of South Carolina, 915 Greene Street, Room 202B, Columbia, SC 29208 USA; 4 College of Medicine, Department of Neurology, Medical University of South Carolina, 96 Jonathan Lucas Street, MSC 606, Charleston, SC 29425-2503 USA; 5 Department of Public Health Sciences, College of Medicine, Medical University of South Carolina, 135 Cannon Street, MSC 835, Charleston, SC 29425-2503 USA; 6 Roxelyn and Richard Pepper Department of Communication Sciences and Disorders, School of Communication, Northwestern University, 70 Arts Circle Drive, Evanston, IL 60208 USA; 7 Feinberg School of Medicine, Otolaryngology – Head & Neck Surgery, Radiation Oncology, Northwestern University, 420 E Superior Street, Chicago, IL 60611 USA; 8 Edward J. Hines Veteran’s Affairs Medical Center, 5000 5th Avenue, Hines, IL 60141-3030 USA

**Keywords:** Pulse rate, Fluoroscopy, Deglutition disorder, Diagnosis

## Abstract

Modified Barium Swallow Studies (MBSS) are a critical part of the evaluation, treatment planning, and outcome assessment for persons with swallowing disorders. Since MBSSs use ionizing radiation with associated cancer risks, many clinicians have reduced radiation exposure by reducing the fluoroscopic pulse rate. However, by reducing pulse rate, we also decrease the temporal resolution of MBSSs which has been shown in pilot studies to significantly reduce diagnostic accuracy. Two hundred MBSSs from patients routinely undergoing MBSS as standard of care conducted at 30 pulses per second (pps) using the Modified Barium Swallow Study Impairment Profile (MBSImP™) standardized administration protocol were selected. A stratified sampling method ensured that a full range of swallowing impairments (etiology, type, and severity) was represented. Recordings were down sampled from 30 pps to 15, 7.5, and 4 pps. MBSSs were rated using the MBSImP components and Penetration–Aspiration Scale (PAS) score for each swallow. Percent agreement was calculated across raters for MBSImP and PAS scores by bolus type and volume. The Least-Squares Method was used for hypothesis testing. Statistically significant and clinically meaningful changes in scores of swallowing physiology and penetration/aspiration occurred when reducing pulse rate below 30pps. These changes were evident across bolus types and volumes. Given the impact on diagnostic accuracy and the low radiation risks to adults undergoing MBSSs, reducing pulse rate to 15pps or below is not aligned with the As Low As Reasonably Achievable (ALARA) principle and should not be used as a viable method to reduce radiation exposure from MBSSs.

## Introduction

The Modified Barium Swallow Study (MBSS) is a key diagnostic tool for the evaluation of swallowing physiology and aides in the determination of treatment plans for adults with known or suspected swallowing impairment. MBSSs are a fluoroscopic examination that utilizes ionizing radiation. Ionizing radiation at the level used in MBSSs does not cause deterministic effects (skin burns, etc.) [1, 2] but could cause stochastic effects since there is no threshold for such effects [3, 4]. The main stochastic effect is cancer. All radiographic studies, including the MBSS, are conducted under the As Low As Reasonably Achievable [5] principle of radiation safety. ALARA simply means the elimination of all unnecessary exposure to radiation, which can be defined as exposure that does not contribute to improved diagnostic performance. Importantly, the ALARA principle dictates that clinically important diagnostic accuracy should not be compromised due to stochastic risks, such as those associated with MBSSs.

Until recently, we did not know that the level of cancer risks associated with MBSSs in adult patients is very low [1, 6–11]. Thus, some clinicians, in an attempt to protect the best interest of their patients, have implemented the practice of reducing the fluoroscopic pulse rate as a method to decrease radiation exposure and associated cancer risks. Pulse rate describes the temporal characteristics (rate) of the emitted radiation beam and is defined as the number of pulses per second (pps). Pulse rates for fluoroscopy are typically able to be set at 30, 15, 7.5, and 4 pps. While reducing pulse rate decreases radiation exposure, it also decreases temporal resolution and diagnostic accuracy. Decreasing pulse rate has a direct and proportional effect on the number of unique images in which a swallow is captured. Since the oropharyngeal swallow only lasts approximately 1 s [12], when pulse rate is decreased from 30 to 15 pps, the number of unique images available to judge swallowing impairment also decreases from 30 to 15 pps [13]. This makes the swallow motion appear less continuous or ‘choppy.’ This decrease in number of images similarly occurs when pulse rates are decreased to 7.5 pps and 4 pps, leading to 7.5 pps and 4 pps images from which to judge swallowing impairment, respectively. The decrease in number of images in which a swallow is captured and consequential reduction in the information available from which swallowing impairment can be judged, is not trivial, because specific physiological components occur in tight temporal synchrony that must be accurately assessed to identify the appropriate physiological targets for treatment [14–18]. These components are only assessable at specific points during the swallow and, thus, may not be captured when using pulse rates lower than 30 pps.

Prior smaller studies have supported the hypothesis that reduction in temporal resolution of MBSSs negatively impacts diagnostic accuracy [13, 19]. Specifically, Bonilha, Blair, and Carnes et al. [13] found that diagnostic accuracy of swallow physiology was negatively impacted in 100% of MBSSs, and PAS was impacted in 80% of MBSSs. This finding is further supported by data detailing the brevity of some aspects of swallow physiology used for defining diagnosis, treatment planning, and treatment outcomes [20]. The consequences of incorrectly assessing swallowing impairment are serious. If swallowing impairment exists and is un- or under-detected, airway protection and nutrition may be at risk. Inaccurate judgments may also err on the side of overly conservative recommendations of oral intake restriction such as modifications in diet or tube-feeding placement that may unnecessarily decrease a dysphagic patient’s health status and quality of life. Thus, a larger, definitive study was needed to provide a sufficient high level of evidence to drive clinical changes.

There were three main aims for this larger, definitive study with associated hypotheses.

- *Aim 1*: Determine the influence of pulse rates of 15 pps or less on the visualization of swallow physiology via MBSS. We hypothesized that the reduction of temporal resolution, associated with reducing the pulse rate, impairs clinician judgments of clinically meaningful aspects of swallowing physiology.
- *Aim 2*: Determine which aspects of swallow physiology are most sensitive to degraded temporal resolution. We hypothesized that the reduction of temporal resolution would have a greater impact on visualization of aspects of swallow physiology that are more rapid than others or that must be assessed at a specific swallow timepoint to reflect anatomical configuration.
- *Aim 3*: Determine which bolus types (bolus volume/viscosity) are most sensitive to degraded temporal resolution. We hypothesized that the reduction of temporal resolution would impact visualization for all bolus types.

## Methods

This study was approved by the Institutional Review Board (IRB). The study was performed retrospectively, sampling from a two hundred MBSSs retrieved from the Clinical Data Warehouse (CDW) at the Medical University of South Carolina. The CDW was used to identify patients who had undergone a Modified Barium Swallow Study (MBSS) as part of their standard of care.

The medical records of the patients were screened to identify those patients with swallowing characteristics that fulfill the study requirements. Demographic and past medical history data were extracted from the electronic health records (EHR) of eligible participants. We aimed to include studies which represented the full range of impairments as well as those that fell into certain diagnostic categories representing the various etiologies of swallowing disorders. Therefore, a stratified sampling method was used to ensure that the included clinical MBSSs represented the full range of swallowing impairments (etiology, physiological impairment, and severity).

### Eligibility Criteria

All eligible participants had an MBSS as part of standard of care and were 21 years or older. All MBSSs were performed at 30 pps and were recorded at 30 fps according to best practices [13, 21]. All MBSSs were conducted using the Modified Barium Swallow Impairment Profile (MBSImP™) [22, 23] which measures 17 distinct physiologic components of swallowing. The core protocol uses 12 swallows across varying liquid and solid consistencies, the initial 10 in the lateral view, and the last 2 in the anterior–posterior view. The standard MBSImP protocol uses standardized commercial preparations of barium contrast agents (Varibar®, Bracco Diagnostics, Inc.): thin liquid barium (two trials of 5 mL thin liquid, one cup sip trial, one sequential swallow trial); nectar-thick liquid barium (one 5 mL trial, one cup sip trial, and one sequential swallow trial), honey-thick liquid barium (one 5 mL trial), pudding-thick barium (one 5 mL trial), and a one-half portion of a Lorna Doone shortbread cookie coated with 3-mL pudding-thick barium). There are also two trials in the A-P view: nectar-thick liquid barium (one 5 mL trial). In addition, the consistencies used in the MBSImP can be translated into compatible International Dysphagia Diet Standardization Initiative (IDDSI) levels [24, 25]. Pudding-thick barium (one 5 mL trial). From these clinical MBSSs, data related to swallowing impairment and swallowing outcomes were extracted. Physiologic swallowing assessment in the form of MBSImP component scores were collected as well as Penetration–Aspiration Scale (PAS) scores [26] for each trial. Clinical assessment scores (MBSImP and PAS) were used to select MBSSs to allow for stratified sampling ensuring that the sample of MBSS used in this study reflected the entire range of scores across all physiological impairments.

*Inclusion criteria* were the use of the MBSImP protocol, recording at 30 pps, lack of significant structural changes or hardware (to minimize recall bias), and fitting a needed diagnostic and impairment severity category for stratification. The original clinical report for each examination was used to stratify the MBSSs by swallowing impairment severity and broad diagnostic category (pulmonary, neurologic, head, and neck cancer). This is important since it is possible that pulse rate will have different effects on MBSSs of severe versus mild swallowing impairments. On the MBSImP, there are 17 individual physiological components of swallowing impairment that are judged and reported on 3-, 4-, and 5-level scales. There﻿ are 4 physiological components that are judged on a 3-level scale, 4 components judged on a 4-level scale, and 9 components judged on a 5-level scale. Thus, 73 levels of swallowing impairment were used for stratification. Specifically, patients were stratified so that each of the 73 levels were represented by 2 patients. The remaining 54 patients were selected based on the distribution of swallowing impairment in the clinical population so that the most common types of swallowing impairments are represented proportionately without excluding the less common types. The sampling continued until 146 MBSSs were identified based on MBSImP individual physiological component stratification with 54 MBSSs representative of the clinical population who undergoes MBSSs.

### Simulating Lower Pulse Rates

**Fig. 1:**
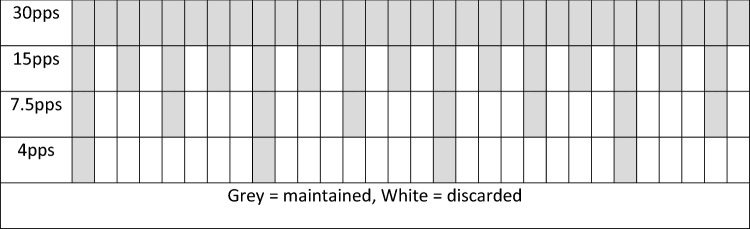
Frames maintained and discarded for each of the 4 pulse rates

Pulse rates lower than 30 pps were simulated, rather than directly captured from the patient MBSSs for two reasons: (1) for accurate assessment of the influence of pulse rate identical swallows need to be captured in the recordings being compared and, (2) conducting MBSSs of patients at a lower pulse rate than we presume to be clinically necessary may be viewed as unethically exposing patients to additional ionizing radiation. MBSSs recorded at 30 pps were downsampled to simulate the different pulse rate conditions at 15 pps, 7.5 pps, and 4 pps. These pulse rates were chosen as they represent the most frequent pulse rates used during MBSSs in the United States [27]. Pulse rate options are set by the fluoroscopy equipment manufacturer. Frames from pulse rates lower than 30 were duplicated to directly replicate the process used by fluoroscopy equipment as set by the manufacturers [28]. This downsampling process also maintained the length of the recording across pulse rates which masked the differences between pulse rate recordings by eliminating duration as a potential confounder (see Fig. 1). For the 15 pps simulations, we extracted every other frame and replaced it with the prior frame. This maintained the frame rate and duration of the recording (30 fps) while providing only the information available from a 15 pps recording. The same method was used to simulate 7.5 pps and 4 pps. To simulate 7.5 pps, after the first frame, every fourth frame was selected and repeated 3 times except for the last frame which was only be repeated one time. To simulate 4pps, the 1st, 9th, 17th, and 25th frames were selected and retained. The 1st, 9th, and 17th frames were repeated 7 times while the 25th frame was repeated 5 times. This process resulted in 800 MBSS recordings (200 patients × 4 pulse rates) for analysis. See Fig. 1 for a graphic representation of the frames which were maintained and repeated (in gray) and the frames that were discarded (in white) for the 4 different pulse rate conditions.

### MBSS Presentation

The MBSSs were presented to the scoring SLPs (raters) at different pulse rates in a randomized order with at least 2 weeks interposed between the SLP raters’ viewing of the swallows at different pulse rates from the same participant. The randomization and time delay were enacted to minimize recall bias. The SLP raters were asked to report if they recognized a recording at the time of presentation. If recognition was reported, the interval between scoring of MBSSs from the same patient was lengthened. SLP raters had access to the MBSS videos during the entire rating period.

### MBSImP Scoring

SLPs from the Medical University of South Carolina were recruited as participant raters to score the MBSS video files individually using the MBSImP methodology [22]. All raters completed the MBSImP training (registered MBSImP clinicians) and passed tests demonstrating at least 80% accuracy [29]. For the purposes of MBSImP, videos are viewed in slow motion and at times frame by frame. This viewing methodology is supported by preliminary findings that demonstrated viewing MBSS in slow motion improves rating accuracy [30]. All recordings were scored by 2 SLPs to ensure that the magnitude of the differences in swallowing severity assessments are attributable to pulse rate as opposed to rater variability. Furthermore, 20% of samples were repeated by the same SLP for assessment of intrarater reliability.

MBSImP component ratings were made for each swallow (swallow-by-swallow rating) by each rater. The individual MBSImP components scores are judged separately on each swallow, are used to decide on treatment strategy, can be used to determine severity of swallowing impairment, and can help elucidate the mechanism by which pulse rate influences MBSImP scores.

### Penetration and Aspiration Scale (PAS) Scoring

The PAS is used to describe “the depth to which material passes in the airway and whether or not material entering the airway is expelled” [26]. The PAS is scored on an ordinal scale between 1 and 8, with one being bolus does not enter airway and 8 is entered in airway below folds with no effort to eject it by the patient. The PAS was judged, and scores were recorded for each swallow in the lateral position.

Discrepancies in MBSImP or PAS scores were handled by convening the two SLPs who rated the recording to review the recording and attempt agreement.

### Data and Statistical Analyses

Generalized linear mixed models (GLMMs) were used to estimate intraclass correlation coefficients (ICCs), quantifying the degree to which variation in impairment scores (i.e., PAS, oral total, pharyngeal total) are attributable to pulse rate, as opposed to swallowing task or subject-to-subject variability. Since the 4 different pulse rates can be tested within subjects’ individual swallows, subjects are essentially able to serve as their own controls. The GLMMs are hierarchical models, allowing us to model the relationship between pulse rate and variation in swallowing severity assessments, using fixed effects for pulse rate and swallow task, and random subject effects (to account for dependence among measurements made on the same subjects). The GLMMs also allowed us to account for differences in impairment scores that are attributable to specific diagnoses (e.g., pulmonary, neurologic, head, and neck). We compared several different error structures by using AIC values to determine the best model fit.

## Results

Two hundred participants’ MBSSs were selected for inclusion in this study. The mean age was 61.6 years (range 21–95), most were white, and 0.5% identified as Hispanic. The majority (60%) were female. Etiologies included head and neck cancer (28/200, 14%), neurologic diagnoses (e.g., stroke, progressive neuromotor disease, dementia) (57/200, 28.5%), pulmonary disorders (12/200, 6%), and diagnoses that fell into an “other” category (103/200, 51.5%). See Table 1.

**Table 1:** Demographics and primary diagnoses of cohort

	Full cohort *n* = 200 *n* (%)
Age (mean, SD, 95% confidence interval)	61.6 years ± 13.77, (59.68, 63.53)
Race (*n*, %)	
White	151 (75.5)
Black	43 (21.5)
Asian	2 (1)
Other	3 (1.5)
Unknown	1 (.5)
Sex (*n*, %)	
Female	120 (60)
Primary diagnosis (*n*, %)	
Head and neck cancer	28 (14)
Neurologic	57 (28.5)
Pulmonary	12 (6)
Cervical spine surgery	18 (9)
Gastroenterology	21 (10.5)
Thyroidectomy	2 (1)
Globus	9 (4.5)
Rheumatologic	4 (2)
Otolaryngology	19 (9.5)
Cardiothoracic surgery	1 (0.5)
Multiple comorbidities	29 (14.5)

### Impact on Impressions of Swallowing Physiology (MBSImP Scores) and Impressions of Airway Invasion (PAS Scores)

Reducing pulse rate had a statistically significant impact on eight components of the MBSImP and on PAS scores. Initiation of pharyngeal swallow (component 6), epiglottic movement (component 10), pharyngeal stripping wave (component 12), and pharyngoesophageal segment opening (component 14) were statistically significantly impacted across bolus types and volumes in the GLMM model. See Table 2 and Appendix A for full details.

**Table 2:** Influence of Reducing Pulse Rate on Selected MBSImP Scores and PAS score

	5 mL thin	Cup sip thin	Sequential thin	5 mL nectar	Cup sip nectar	Sequential nectar	5 mL Honey	5 mL Pudding	1/3 Lorna Doone with 3 mL Pudding
Oral residue (Component 5)	0.0458				0.0365				
Initiation of pharyngeal swallow (Component 6)	0.0001	0.0001	0.0001	0.0001	0.0001	0.0001	0.0001	0.0001	0.0001
Velar elevation (Component 7)		0.0128				0.0257			
Laryngeal elevation (Component 8)						0.0018			
Epiglottic movement (Component 10)	0.0001	0.0013	0.0002	0.0001	0.001	0.0314	0.0001	0.0012	0.0013
Pharyngeal stripping wave (Component 12)	0.0001	.0001	.0001	.0001	.0001	.0001	.0001	.0001	.0001
Pharyngoesophageal Segment opening (Component 14)	0.004	0.0001	0.0001	0.0004	0.0024	0.0304	0.0044	0.0001	0.0074
Base of tongue retraction (Component 15)		0.0493	0.0113		0.0131	0.0009		0.0272	0.0194
PAS score				0.0239	0.04	0.0162	0.014	0.0001	0.004

General linear models were used to determine statistically significant relationships

Data were also analyzed for clinical significance using the metric of influence on rater reliability. Rater reliability decreased systematically with reduction of pulse rate, confirming the validity of rater reliability as a measure of difficulty in making judgments regarding MBSImP and PAS scores (Table 3). Eight of the 17 MBSImP components (47%) were moderately or severely impacted when assessed at a pulse rate of 15, increasing to 12 out of 17 (70%) components at a pulse rate of 7.5 pps, and 16 out of 17 (94%) components at a pulse rate of 4 pps. PAS was also moderately to severely impacted by pulse rate modifications. In fact, 12 of the 17 aspects of swallowing physiology (MBSImP components) were impacted from a reduction in pulse rate from 30 to 15 pps.

**Table 3:** Influence of reducing pulse rate on agreement across MBSImP components and PAS scores

Physiologic component	30 vs. 15 pps	30 vs. 7.5 pps	30 vs. 4 pps
Lip closure (Component 1)	Moderate-severe	Moderate-severe	Severe
Tongue control during bolus hold (Component 2)	Moderate	Moderate	Moderate/severe
Bolus preparation/mastication (Component 3)	Moderate-severe	Severe	Severe
Bolus transport/lingual motion (Component 4)	Moderate	Moderate/severe	Severe
Oral residue (Component 5)	Mild	Mild	Moderate
Initiation of pharyngeal swallow (Component 6)	Severe	Severe	Severe
Soft palate elevation (Component 7)	Not impacted	Not impacted	Not impacted
Laryngeal elevation (Component 8)	Mild	Moderate	Severe
Anterior hyoid excursion (Component 9)	Moderate	Moderate	Severe
Epiglottic movement (Component 10)	Not Impacted	Mild	Moderate-severe
Laryngeal vestibular closure (Component 11)	Not Impacted	Not impacted	Moderate
Pharyngeal stripping wave (Component 12)	Moderate	Severe	Severe
Pharyngeal contraction (Component 13)	Not impacted	Mild	Moderate-severe
Pharyngoesophageal segment opening (Component 14)	Mild	Moderate	Severe
Tongue base retraction (Component 15)	Mild	Moderate	Severe
Pharyngeal residue (Component 16)	Not Impacted	Moderate	Moderate
Esophageal clearance (Component 17)	Moderate	Moderate/severe	Moderate
Penetration–aspiration scale (PAS)	Moderate	Moderate	Severe

Severe: ≤ 70% agreement across all raters; Moderate-Severe: ≤ 70% agreement across subset of raters; Moderate: ≤ 80% agreement across all raters, Mild: ≤ 80% agreement across a subset of raters; Not Impacted: ≥ 80% agreement across all raters

### Impact on Judgments by Bolus/Trial Type

The impact of reducing pulse rate from 30 to 15 pps was seen across all bolus/trial types. See Tables 2 and 4. Our unadjusted analysis showed that judgments for laryngeal elevation (component 8), epiglottic movement (component 10), pharyngeal stripping wave (component 12), and PAS were statistically significantly different when comparing 7.5 pps to 30 pps.

**Table 4:** Impact of reducing pulse rate across bolus types

Bolus type	30 vs. 15 pps	30 vs. 7.5 pps	30 vs. 4 pps
Second 5 mL thin liquid	Moderate	Moderate	Severe
Cup sip thin liquid	Moderate	Moderate	Severe
Sequential sips thin liquid	Moderate	Moderate	Severe
5 mL nectar-thick liquid	Mild	Moderate	Severe
Cup sip nectar-thick liquid	Mild	Moderate	Severe
Sequential sips nectar-thick liquid	Moderate	Moderate	Severe
5 mL honey-thick liquid	Mild	Moderate	Severe
5 mL Pudding	Mild	Moderate	Severe
1/3 Cookie with 3 mL pudding	Mild	Moderate	Severe

Severe: ≤ 70% agreement across all raters; Moderate-Severe: ≤ 70% agreement across subset of raters; Moderate: ≤ 80% agreement across all raters, Mild: ≤ 80% agreement across a subset of raters; Not Impacted: ≥ 80% agreement across all raters

## Discussion

The results reflect that reducing the pulse rate impacts the evaluation of key aspects of swallowing physiology and the assessment of airway penetration/aspiration. This impact occurs across bolus types and volumes. These results, paired with our knowledge of the low cancer risks associated with MBSSs in adults, indicate that pulse rate should not be reduced (to 15 pps or below) for any trial type during an MBSS.

### Impact of Pulse Rate on Swallowing Physiology and Penetration/Aspiration

The results of this larger, definitive study support previously published results from a pilot investigation and show that the temporally dependent nature of swallowing movements and bolus flow necessitate fluoroscopy settings that allow for sufficient temporal resolution (greater than 15pps). Reducing pulse rate to 15 pps or below has an impact on the identification of swallowing physiology and penetration/aspiration during MBSSs. Specifically, Bonilha, Blair, Carnes, et al. [13], identified differences between 30 and 15 pps in six physiological aspects of swallowing (initiation of pharyngeal swallow (component 6), anterior hyoid excursion (component 9), epiglottic movement (component 10), pharyngeal contraction (component 13), pharyngoesophageal segment opening (component 14), and tongue base retraction (component 15)). In comparison, in this larger study, we identified differences in 12 out of 17 physiological aspects of swallowing with the differences in eight of those aspects being moderate to severe. Both Bonilha, Blair, Carnes et al. [13] and the current study identified PAS scores as being impacted by decreasing pulse rate from 30 to 15 pps. In a cohort of 20 ischemic stroke patients, Mulheren et al. [19] found reducing MBSS pulse rate from 30 to 15 pps impacted measures/scores of pharyngeal transit time, oral residue, pharyngoesophageal segment opening, bolus transport, and initiation of pharyngeal swallow. Thus, all studies to date support the conclusion that reducing pulse rate diminishes diagnostic accuracy.

### Impact of Pulse Rate across Bolus Types and Volumes

Many centers have used an approach of reducing pulse rate for select boluses with the hypothesis that thicker boluses would be impacted less by a reduction in temporal resolution than thinner boluses. In this study, we found that while thinner liquids were more impacted by pulse rate reduction when pulse rate was reduced from 30 to 15 pps, all bolus types and volumes were at least mildly impacted. Due to the minimal additional cancer risk related to conducting MBSSs in adults at 30pps, as well as the now known impact on diagnostic accuracy, it goes against the ALARA principle to reduce pulse rates for any bolus type. Thus, it is not recommended that pulse rate be reduced based on rheologic properties of bolus or bolus volume.

### Additional Cancer Risks due to Radiation Exposure for Adults Undergoing MBSS are Very Low

In addition to degradation in diagnostic accuracy, we now have a better understanding of radiation exposure and associated cancer risks for adults undergoing MBSSs. Bonilha et al. [8] identified that radiation exposure was an average of 0.27 mSv per exam (for perspective, this is less than the amount of radiation emitted from a person’s body in a year) [1, 6, 10]. This radiation exposure is less than that from a mammogram (0.4 mSv) and approximately 1/8th that from a head CT (2 mSv) [4, 31]. The MBSS-associated cancer incidence risk ranged from 0.0032% for a 20-year-old female, the highest risk group, to 0.00049% for a 60-year-old male, the lowest risk group. When paired with conservative US cancer incidence data that indicates 33% of the population will have a diagnosis of cancer in their lifetime, these values indicate an extremely low increased cancer incidence risk of 0.0097% for a 20-year-old female with lower increased risks for males or older individuals [1]. Thus, degrading diagnostic accuracy by reducing pulse rate is not an evidence-based clinical practice and is not in line with the ALARA principle [1, 9, 13, 14, 17, 27].

### Implications for Adult Populations

Whenever possible, SLPs should advocate for MBSSs to be conducted at pulse rates above 15 pps. If a pulse rate above 15 pps is not available on a specific fluoroscopic unit, the continuous beam mode should be used. If it is not possible to acquire MBSSs in pulse rates above 15pps or in continuous mode, SLPs and other clinicians relying on the information provided by the MBSS must be aware of the limitations of the imaging. For example, SLPs can be confident that if they visualize penetration or aspiration than it occurred; however, the extent (volume or location) of the penetration or aspiration cannot be relied upon for clinical decision making as critical frames to determining volume and location will be missing. Similarly, information from *some* components of the MBSImP is achievable at rates of 15 pps; however, this is also limited, and extent of impairment will not be able to be ascertained. Some MBSImP components should not be judged from MBSSs achieved with pulse rates of 15 pps or less as these components must be judged at a specific timepoint in the swallow and lower sampling rates do not allow for valid identification of the timepoint (i.e., maximum excursion of the hyoid, etc.). We recommend acknowledging this limitation in the MBSS documentation with a disclaimer stating the diagnostic limitations in the interpretation of swallow function from MBSSs performed at 15pps or lower.

### Implications for Pediatric Populations

While we cannot apply radiation exposure or cancer risk findings from adults to children, we do have information to be able to apply the pulse rate findings from adults to children. The length of the upper aerodigestive tract in a young child is approximately half the size of an average adult and a young child’s swallow occurs in approximately one third the time when compared to that of an adult swallow. Specifically, the length of an infant’s vocal tract is between 7 and 8 cm (cm) while an adult’s vocal tract is between 15 and 18 cm [8]. This difference in length partially accounts for the significantly reduced pharyngeal transit time in infants, reported to be approximately0.27 s (s) [8], when compared to that in adults, approximately 0.91 s [7]. Thus, the number of images available to detect a swallowing impairment is reduced by more than half and, therefore, it can be inferred that the diagnostic accuracy of a recording at 30 pps in a young child would be worse than that of a MBSS recorded at 15 pps in an adult. However, a large, definitive study on the influence of pulse rate on diagnostic accuracy of bottle-fed infants is on-going to directly address this question.

### Limitations

The study was accomplished using retrospectively collected data which was necessary to acquire a stratified sample of clinically indicated MBSSs during the study period.

## Conclusion

Our findings unequivocally demonstrate that pulse rates of 15 pps or lower influence clinicians’ ability to accurately visualize critical physiological aspects of the swallow and their observations of penetration and aspiration. When paired with the knowledge that radiation exposure and related cancer risks to adults undergoing MBSS is very low, clinicians should use the information gained from this study to advocate for only performing MBSSs with pulse rates above 15pps across all bolus types.

## Figures and Tables

**Table 5:**
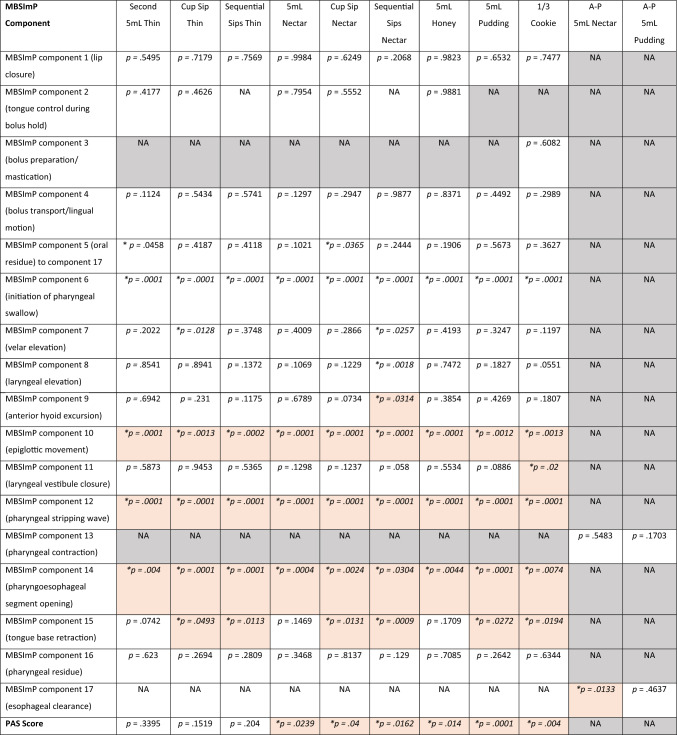
Comparison of measures of swallowing physiology across four different pulse rates

Modified Barium Swallow Impairment Profile (MBSImP™©), Anterior–Posterior (A-P), Penetration-Aspiration Scale (PAS); P-values were obtained using general linear mixed models and were not adjusted for multiple comparisons; *p < *.05*

## Data Availability

Data can be made available upon reasonable request to corresponding author.

## Appendix

See Table ^5^
